# Metabolic Interactions in the Tumor Microenvironment of Classical Hodgkin Lymphoma: Implications for Targeted Therapy

**DOI:** 10.3390/ijms26157508

**Published:** 2025-08-04

**Authors:** Michał Kurlapski, Alicja Braczko, Paweł Dubiela, Iga Walczak, Barbara Kutryb-Zając, Jan Maciej Zaucha

**Affiliations:** 1 Department of Hematology and Transplantology, University Clinical Centre, Medical University of Gdansk, 80-211 Gdańsk, Poland; mkurlapski@uck.gda.pl; 2 Department of Biochemistry, Medical University of Gdansk, 80-211 Gdańsk, Poland; alicja.braczko@gumed.edu.pl (A.B.); igawalczak@gumed.edu.pl (I.W.); 3 Department of Regenerative Medicine and Immune Regulation, Medical University of Bialystok, 15-269 Białystok, Poland; paweldubiela89@gmail.com; 4 Centre for Experimental Cardiooncology, Medical University of Gdansk, 80-211 Gdańsk, Poland

**Keywords:** Hodgkin lymphoma, cell metabolism, therapeutic strategies, tumor microenvironment

## Abstract

Classical Hodgkin lymphoma (cHL) is a biologically and clinically unique malignancy characterized by rare Hodgkin and Reed–Sternberg (HRS) cells surrounded by a dense and diverse inflammatory infiltrate. These malignant cells actively reshape the tumor microenvironment (TME) through metabolic reprogramming and immune evasion strategies. This review synthesizes current knowledge on how metabolic alterations contribute to tumor survival, immune dysfunction, and therapeutic resistance in cHL. We discuss novel therapeutic approaches aimed at disrupting these processes and examine the potential of combining metabolic interventions with immune-based strategies—such as immune checkpoint inhibitors (CPIs), epigenetic modulators, bispecific antibodies, and CAR-T/CAR-NK cell therapies—which may help overcome resistance and enhance anti-tumor responses. Several agents are currently under investigation for their ability to modulate immune cell metabolism and restore effective immune surveillance. Altogether, targeting metabolic vulnerabilities within both tumor and immune compartments offers a promising, multifaceted strategy to improve clinical outcomes in patients with relapsed or refractory cHL.

## 1. Introduction

In 1865, Samuel Wilks first coined the term Hodgkin’s disease to describe a case involving enlarged lymph nodes and spleen, building upon the foundational work of Thomas Hodgkin and thus immortalizing the name of the original investigator [1]. Subsequent histopathological studies by Wilks and others confirmed that this condition was distinct from other causes of lymphadenopathy, establishing it as a unique clinical and pathological entity [1]. Over the following decades, progressive advances in pathology and microscopy further refined the understanding of this disease. The distinctive multinucleated cells that characterize this disease were independently described by Carl Sternberg in 1898 and Dorothy Reed in 1902, leading to the term Reed–Sternberg cells that remains pathognomonic for classical Hodgkin lymphoma (cHL) today. Their discovery helped dispel the prevailing notion that Hodgkin’s disease was merely a form of tuberculosis, as both conditions commonly present with similar constitutional symptoms including night sweats, weight loss, fever, and lymphadenopathy [2,3]. The development of histological subtypes and the advent of immunohistochemistry in the 20th century revolutionized its classification, ultimately leading to the modern designation of Hodgkin lymphoma [4].

cHL is a unique neoplasm in which cancerous Hodgkin and Reed–Sternberg (HRS) cells constitute only a small fraction of the tumor mass. These malignant cells are surrounded by an abundant and heterogeneous inflammatory infiltrate—including B and T lymphocytes, plasma cells, macrophages, histiocytes, granulocytes, eosinophils, mast cells, mesenchymal stromal cells, and endothelial cells—recruited and modulated by the HRS cells [5].

The evolution of cHL research has been marked by several pivotal discoveries that have transformed our understanding of disease pathogenesis. Early studies established the B-cell origin of HRS cells through genetic analysis, revealing immunoglobulin heavy- and light-chain V gene rearrangements specific to germinal center-experienced B cells [6,7]. The identification of constitutive NF-κB pathway activation as critical for HRS cell survival represented a breakthrough in understanding the molecular basis of the disease [8]. More recently, the discovery of 9p24.1 chromosomal amplification leading to overexpression of PD-L1 and PD-L2 has provided the rationale for immune checkpoint inhibitor therapy, marking a paradigm shift toward immunotherapy-based approaches [9,10].

The precise mechanisms by which HRS cells regulate their microenvironment—and how the tumor microenvironment (TME), in turn, supports and sustains these cells—remain incompletely understood. Although cHL was first identified nearly two centuries ago, it remains an area of active research, particularly regarding the interactions between malignant and stromal cells. Advancements in this area have paved the way for novel therapeutic strategies, including immune checkpoint inhibitors (CPIs) (e.g., PD-1, CTLA-4), inhibitors of signaling pathways such as mTOR and PI3Kδ, CD30 antibody–drug conjugates, monoclonal antibodies (e.g., anti-LAG-3, anti-CD47-signal regulatory protein), immunomodulatory drugs, and cellular therapies such as chimeric antigen receptor-engineered T cells (CAR-T) and chimeric antigen receptor-engineered natural killer cells (CAR-NK). Many of these therapies exert their effects by targeting or modulating the TME [8,11,12,13].

The prognosis for cHL has improved dramatically since the introduction of effective combination chemotherapy regimens. The development of MOPP (mechlorethamine, vincristine, procarbazine, and prednisone) in the 1960s achieved overall survival rates exceeding 50%, which were further improved with the introduction of ABVD (doxorubicin, bleomycin, vinblastine, and dacarbazine) to more than 65% [14]. Contemporary treatment approaches, incorporating risk-adapted strategies and novel agents, have achieved 5-year overall survival rates exceeding 90% in many patient cohorts. This remarkable evolution has established cHL as a pivotal success story in oncohematology, demonstrating the potential for highly effective cancer therapies [14]. cHL is a rare lymphoma, accounting for approximately 10% of all lymphoma cases and 0.5% of all malignancies, with an annual incidence of 2–3 cases per 100,000 individuals [15]. It displays a bimodal age distribution, with a first peak in the third to fourth decades of life and a second peak in the seventh decade, reflecting a higher incidence in older adults. These age-related variations underscore the need for treatment optimization. In younger patients, the focus is on minimizing long-term adverse effects, such as infertility and secondary malignancies. In older patients, reducing early treatment-related toxicity is paramount [16]. The development of targeted therapies continues to expand, aiming to reduce the toxicity of conventional chemotherapy and radiotherapy while improving the efficacy, particularly in high-risk cHL patients.

In this comprehensive review, we aim to elucidate the intricate metabolic pathways that underlie the pathophysiology of classical cHL and explore how these pathways can be exploited for therapeutic intervention. By examining recent research on metabolic dependencies and vulnerabilities in cHL, we seek to identify novel therapeutic targets that could complement or enhance current treatments, including CPIs and targeted therapies. This review highlights recent discoveries in cancer metabolism. It provides a detailed analysis of how these findings may inform innovative treatment strategies for cHL, to improve patient outcomes and minimize the adverse effects of standard therapies.

## 2. Tumor Microenvironment (TME) in cHL

### 2.1. Composition of the TME

cHL is distinguished by a unique TME where the malignant HRS cells represent only a small fraction—typically about 1–5%—of the total tumor mass [12,17,18]. The vast majority of the TME consists of a diverse array of non-malignant cells, including various immune infiltrates (such as T cells, B cells, plasma cells, macrophages, eosinophils, neutrophils, mast cells, and natural killer cells) and stromal components (fibroblasts and extracellular matrix) [19]. Among immune cells, CD4+ T cells are especially prominent, often forming rosettes around HRS cells [20]. Quantitative analysis of the TME reveals that T lymphocytes represent the most numerous cell type, with a median proportion of 55% (range from 1.1% to 83%) of total tumor cellularity [21]. Among these, CD4+ T cells are the most abundant subset, comprising a median of 25% (range from 0.5% to 48%) of tumor cellularity and often forming characteristic rosettes around HRS cells [20,21]. CD8+ cytotoxic T lymphocytes constitute a median of 7.4% (range from 0.2% to 34%), while regulatory T cells represent 1.7% (range from 0% to 20%) of the cellular composition [21].

Tumor-associated macrophages constitute approximately 20% (range from 8.0% to 49%) of tumor cellularity. The remaining cellular components include B cells, plasma cells, eosinophils, neutrophils, mast cells, and natural killer cells, each contributing smaller but functionally important fractions to the overall TME architecture [21].

Notably, the TME exhibits distinct compositional patterns, with an inverse correlation between T cell and TAM proportions (rs = −0.45; *p* < 0.001), suggesting that cHL tumors can be broadly categorized into T cell-predominant or macrophage-predominant microenvironments [21]. This compositional heterogeneity has significant implications for treatment response and prognosis, as different cellular compositions may respond differently to various therapeutic interventions. The composition and density of these infiltrates can vary depending on the histological subtype of cHL, such as nodular sclerosis or mixed cellularity.

### 2.2. Immune Evasion and Inflammation in cHL Progression

HRS cells in cHL employ a multifaceted array of mechanisms to evade immune detection and clearance, ensuring their survival and proliferation despite being surrounded by a robust but ineffective immune infiltrate. A central strategy involves the overexpression of immune checkpoint ligands PD-L1 and PD-L2 on the surface of HRS cells, primarily due to genetic alterations such as copy number gains of the 9p24.1 chromosomal region, which encodes these proteins [22]. This overexpression leads to the engagement of the PD-1 receptor on T cells, resulting in the inhibition of cytotoxic T-cell activity and the creation of an immunosuppressive TME [22,23]. Additionally, HRS cells frequently harbor mutations in the β2-microglobulin gene, leading to a loss of MHC class I expression, which impairs antigen presentation to CD8+ cytotoxic T cells and further diminishes immune recognition [24]. Some cases also exhibit aberrations in the CIITA gene, which reduces MHC class II expression and limits interactions with CD4+ T-helper cells [25]. Beyond these genetic alterations, HRS cells secrete a range of immunosuppressive cytokines and molecules—including TGF-β, IL-10, macrophage migration inhibitory factor (MIF), and galectin-1—that modulate the surrounding immune cells, promote the recruitment and activation of regulatory T cells (Tregs), and foster an anti-inflammatory, tumor-supportive milieu [25]. HRS cells also express additional surface molecules, such as CD95 ligand [26], HLA-G, HLA-E, and CD200, which can induce apoptosis or functional exhaustion in cytotoxic lymphocytes and natural killer (NK) cells, further blunting anti-tumor immune responses [9]. In cases associated with Epstein–Barr Virus (EBV), viral proteins like LMP1 can upregulate PD-L1 and PD-L2 expression through the activation of JAK/STAT and AP-1 signaling pathways, making EBV-positive HRS cells equally adept at immune evasion as their EBV-negative counterparts [27,28,29]. Collectively, these complementary and overlapping mechanisms allow HRS cells to orchestrate a microenvironment that not only protects them from immune attack but also actively suppresses effective anti-tumor immunity, underpinning the persistence and progression of cHL [30].

## 3. Metabolic Alterations in the cHL Microenvironment

### 3.1. Warburg Effect: Glycolysis vs. Oxidative Phosphorylation in HRS Cells

In cHL, the metabolic landscape of the TME is defined by a striking heterogeneity between the malignant HRS cells and the surrounding non-malignant immune and stromal cells [31,32,33]. Traditionally, many cancers are characterized by the Warburg effect, a phenomenon in which tumor cells preferentially utilize glycolysis for energy production even in the presence of oxygen, resulting in increased glucose uptake and lactate production [34]. This metabolic reprogramming was initially thought to result from dysfunctional mitochondria, but advances in technology have revealed that cancer cells possess functional mitochondria, and the Warburg effect is not consistent across all tumor types [35].

However, emerging evidence indicates that HRS cells in cHL deviate from this paradigm [36]. These cells exhibit high mitochondrial metabolism and rely predominantly on oxidative phosphorylation rather than glycolysis for their energy needs [37].

HRS cells exhibit a pronounced shift toward mitochondrial metabolism, as indicated by increased mitochondrial mass, upregulation of key oxidative phosphorylation (OXPHOS) proteins, and enhanced mitochondrial biogenesis [36]. This metabolic adaptation is particularly striking given that many cancer cell lines, when cultured under physiologically relevant glucose concentrations (1–5 mM rather than the typical 25 mM used in laboratory studies), show increased reliance on mitochondrial respiration rather than glycolysis. Second, the reverse Warburg effect in cHL involves a metabolic coupling mechanism where oxidative stress induced by HRS cells leads to mitochondrial dysfunction in cancer-associated fibroblasts (CAFs). Hydrogen peroxide secreted by HRS cells induces mitophagy and autophagy in surrounding fibroblasts, causing them to lose mitochondrial function and switch from aerobic metabolism to glycolysis [35]. This is supported by elevated expression of mitochondrial proteins such as monocarboxylate transporter 1 (MCT1), which facilitates efficient mitochondrial respiration and lactate import. Studies have shown that the ratio of oxidative to nonoxidative energy conversion in HRS cells is significantly higher than in their regular germinal center B-cell counterparts, with OXPHOS-linked ATP synthesis dominating their energy production. This metabolic adaptation is supported by the activity of transcription factors, such as NF-κB, which promotes the expression of genes involved in mitochondrial function and biogenesis [38,39]. Functionally, both the growth and viability of HRS cells are highly dependent on OXPHOS, and these cells display very low lactate production—a stark contrast to the robust lactate secretion observed in other glycolytic tumors—while simultaneously upregulating the lactate importer MCT1, suggesting that HRS cells may utilize lactate produced by different cells within the TME. This creates a metabolic symbiosis, sometimes referred to as the “reverse Warburg effect,” where stromal and immune cells in the microenvironment engage in glycolysis, exporting lactate via MCT4, which is then imported and metabolized by HRS cells to fuel the tricarboxylic acid cycle and sustain high OXPHOS activity [9,37,40,41].

This compartmentalization of metabolic pathways not only supports the energetic and biosynthetic demands of HRS cells but also contributes to drug resistance, as high OXPHOS activity is associated with increased mitochondrial turnover and autophagy, maintaining mitochondrial quality and resilience under stress. Thus, while the Warburg effect remains a hallmark of many cancers, the metabolic landscape of cHL is defined by a unique interplay between glycolytic and oxidative pathways, with HRS cells leveraging OXPHOS as their principal energy source, supported by the glycolytic activity of their microenvironment [42].

### 3.2. Lipid and Amino Acid Metabolism in Tumor Survival

Lipid and amino acid metabolism play critical and multifaceted roles in lymphoma biology, influencing both malignant cell survival and TME function. In lymphomas, malignant cells frequently upregulate enzymes involved in de novo lipogenesis, particularly fatty acid synthase (FASN), which catalyzes the synthesis of palmitic acid—a precursor for complex lipids essential for membrane biosynthesis, energy storage, and signaling molecules that modulate oncogenic pathways including PI3K, MAPK, and NF-κB [43,44]. This lipogenic activity requires substantial input from glycolytic and pentose phosphate pathway intermediates. It is further enhanced under hypoxic conditions, where hypoxia-inducible factors (HIFs) drive FASN expression to support both glycolysis and oxidative phosphorylation.

Altered lipid metabolism affects not only tumor cells but also the function of immune cells within the TME. For instance, increased lipid accumulation in natural killer (NK) cells within the lymphoma microenvironment impairs their effector responses and metabolism, contributing to immune evasion [45,46]. In lipid-rich conditions, NK cells upregulate peroxisome proliferator-activated receptor gamma (PPAR-γ), which partially restores some mitochondrial functions but ultimately leads to decreased anti-tumor activity [45]. However, direct evidence regarding lipid metabolism in cHL remains limited and requires further investigation.

Amino acids play a crucial role in cancer cell growth and proliferation, supporting key metabolic and signaling pathways. Although amino acid metabolic reprogramming is a characteristic of cancer cells in general, the specific alterations vary by tumor type and depend on factors related to both the tumor and its microenvironment. This metabolic diversity encompasses all aspects of amino acid metabolism, including transporter activity, macromolecule biosynthesis, and nutrient catabolism. Additionally, crosstalk between cancer cells and the TME increases the context-dependent complexity of amino acid metabolism [47]. Wang et al. highlighted the pivotal role of amino acids in hematologic malignancies by summarizing novel therapies targeting amino acid pathways [48,49]. However, a review of the literature reveals a significant gap in research specific to cHL.

Romano et al. confirmed that amino acids play a crucial role in cHL metabolism. In an in vitro model, arginine deprivation resulted in increased uptake of free fatty acids from the medium, thereby maintaining membrane integrity. This adaptive response was associated with elevated oxidative stress, evidenced by decreased levels of glutathione and gamma-glutamylcysteine, and increased levels of cystine and methionine sulfoxide [49]. Metabolomic analysis of plasma from cHL patients revealed elevated levels of hypoxanthine (a final product of purine metabolism) and glycine [50]. The authors suggest that increased glycine concentration may result from impaired cellular uptake of glycine and excessive activity of serine hydroxymethyltransferase, the enzyme responsible for converting serine into glycine [50].

### 3.3. Oxidative Stress in HL

Oxidative stress, characterized by an imbalance between the production of reactive oxygen species (ROS) and the body’s antioxidant defenses, plays a significant role in the pathogenesis and progression of cHL [51]. Studies have consistently shown increased oxidative stress in patients with cHL [52,53]. This is evident in both the malignant HRS cells and the surrounding reactive cellular infiltrates within lymphoma tissue [52]. Elevated markers of oxidative stress have also been detected in the peripheral blood of treatment-naive cHL patients, indicating systemic involvement [52,53]. Marini et al. have identified pronounced mitochondrial and endoplasmic reticulum (ER) redox stress in peripheral blood mononuclear cells from patients with cHL. This oxidative imbalance is more significant in cHL compared to non-Hodgkin lymphoma (NHL), suggesting a disease-specific feature [53]. In cHL, imbalances in pro-oxidant and antioxidant signaling can disrupt normal metabolic processes and cell signaling, contributing to genomic instability, inflammation, and disease progression. Mechanistically, ROS modulate cell signaling and fate, and their dysregulation can disrupt metabolic processes, promote inflammation, and drive disease progression [54]. Additionally, the oxidative damage in cHL is not limited to the TME but may also affect normal tissues, reflecting a systemic inflammatory and oxidative response [53]. Overall, oxidative stress plays a central role in HL pathogenesis, influencing both local and systemic disease features and potentially impacting therapeutic response [53].

### 3.4. Hypoxia and Metabolic Stress Response

The cHL microenvironment is often characterized by hypoxia, acidity, and nutrient deprivation, all of which impose significant metabolic stress on both malignant and non-malignant cells [55,56,57,58]. The expression and activity of key signaling molecules such as ID2, NOTCH1, AP-1, NFκB, and the JAK/STAT pathway—all characteristic features of HRS cells—are upregulated in response to hypoxia in various cell types [55,59,60]. In addition, hypoxic conditions in cancer often lead to a general dedifferentiation of cells, a process in which ID2 and NOTCH1 play significant roles [55]. As a result, several aspects of the HRS cell phenotype closely mirror those observed in hypoxic cells. The central mediator of the cellular response to low oxygen is the HIF-1. HIF-1 consists of an oxygen-sensitive α-subunit and a constitutively expressed β-subunit [61]. Under normal oxygen levels, HIF-1α (or its related protein HIF-2α) is continuously synthesized but is rapidly hydroxylated by prolyl hydroxylases, marking it for polyubiquitination and subsequent degradation. However, when oxygen levels drop and hydroxylase activity decreases, HIF-1α and HIF-2α accumulate in the cell. Upon dimerizing with HIF-1β, HIF-1 becomes an active transcription factor, orchestrating cellular adaptation to hypoxia [62].

Hypoxic conditions, resulting from rapid tumor growth and inadequate vascularization, drive the stabilization of HIFs in HRS cells and other components of the TME [57]. HIF activation upregulates glycolytic enzymes and glucose transporters, further enhancing glycolysis in non-malignant cells such as tumor-associated macrophages (TAMs), while also promoting angiogenesis and adaptation to low-oxygen environments [63,64,65]. Additionally, hypoxia and metabolic stress can induce autophagy and other survival pathways in HRS cells, enabling them to withstand harsh conditions and resist therapy [66,67,68,69]. The interplay between hypoxia, metabolic reprogramming, and immune modulation likely contributes to HL progression by creating a microenvironment that supports tumor cell survival and reduces immune-mediated clearance.

In summary, metabolic alterations in the HL microenvironment are defined by a complex interplay between oxidative phosphorylation in HRS cells, and glycolysis in immune infiltrates, all of which are further shaped by hypoxia and metabolic stress. These adaptations not only sustain tumor growth and survival but also contribute to immune evasion and resistance to therapy.

## 4. Therapeutic Targeting of Glycolytic Metabolism in cHL

**Figure 1:**
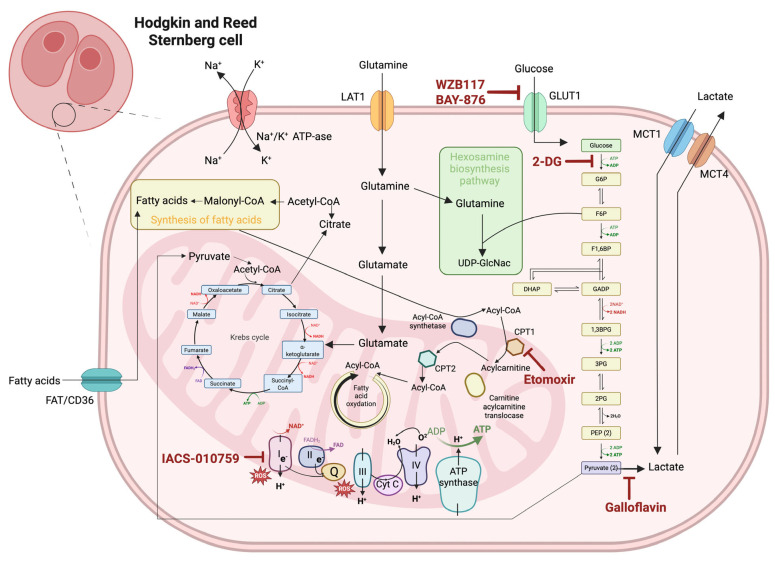
Potential therapeutic targets and agents modulating glucose, fatty acid, and amino acid metabolism in classical Hodgkin lymphoma (cHL). LAT1—L-type amino acid transporter 1, MCT 1 and 4—monocarboxylate transporter 1 and 4, 2-DG—2-deoxy-D-glucose, GLUT1 and 3—glucose transporter 1 and 3, CPT1 and 2—carnitine palmitoyltransferase 1 and 2, UDP-GlcNAc—uridine diphosphate N-acetylglucosamine, ATP—adenosine-5′-triphosphate, ADP—adenosine-5′-diphosphate, Na^+^—sodium ion, and K^+^—potassium ion.

The unique metabolic profile of HRS cells in cHL, characterized by enhanced glycolysis, glutaminolysis, and lipid metabolism, presents several targetable vulnerabilities that are being explored for therapeutic intervention. These approaches aim not only to impair HRS cell survival directly but also to modulate the TME to enhance anti-tumor immunity [70]. An overview of potential therapeutic targets within these metabolic pathways, along with selected promising agents, is provided in Figure 1, which illustrates the key metabolic processes including glucose transport via GLUT1 and GLUT3, glycolytic inhibition by 2-deoxyglucose (2-DG), fatty acid metabolism through carnitine palmitoyltransferase 1/2 (CPT1/2), and amino acid transport via L-type amino acid transporter 1 (LAT1), demonstrating the interconnected nature of these targetable pathways.

### 4.1. Glycolysis Inhibitors

One of the most promising approaches in targeting glucose metabolism in cHL is the use of glycolysis inhibitors. 2-DG, a glucose analog that inhibits hexokinase (the first enzyme in the glycolytic pathway), has shown significant potential in preclinical studies [71]. The mechanism of action of 2-DG involves the competitive inhibition of glucose phosphorylation, leading to the depletion of ATP and subsequent metabolic stress in cancer cells that rely heavily on glycolysis [35]. Importantly, the efficacy of 2-DG appears to be enhanced under physiologically relevant glucose concentrations (1–5 mM) compared to the high glucose conditions (25 mM) typically used in laboratory studies, as cancer cells show greater reliance on mitochondrial respiration when glucose availability is limited [72]. 2-DG not only impairs energy production in HRS cells but also sensitizes them to standard chemotherapy and radiation therapy [73]. By disrupting the Warburg effect, 2-DG reduces lactate production, potentially alleviating the immunosuppressive effects of lactate in the TME [74].

Recent preclinical studies have demonstrated that combining 2-DG with other therapies can enhance its efficacy. For instance, the combination of 2-DG with PI3K/AKT pathway inhibitors has shown synergistic effects in lymphoma cell lines, suggesting a potential strategy to overcome the metabolic plasticity of cancer cells [75]. Recent research has focused on how targeting glucose metabolism in HRS cells can reprogram the TME to improve anti-tumor immunity. The glycolysis inhibitor 2-DG has been shown to enhance the efficacy of PD-1 blockade in preclinical models by alleviating the immunosuppressive effects of lactate in the TME [76]. From a safety perspective, the combination of 2-DG with PI3K/AKT inhibitors requires careful dose optimization. While 2-DG is generally well-tolerated as a single agent, with main side effects including mild gastrointestinal symptoms and transient hyperglycemia, the addition of PI3K/AKT inhibitors can potentiate specific toxicities. PI3K/AKT inhibitors are associated with hyperglycemia, insulin resistance, and potential effects on normal tissue metabolism. However, the combination may allow for lower doses of each agent while maintaining efficacy, potentially reducing overall toxicity burden [77].

One of the key intrinsic factors driving T-cell differentiation and function is their metabolic commitment. It has been demonstrated that dependence on glycolysis regulates the effector response of T cells and leads to the generation of terminal effector T cells [78,79]. Moreover, in studies conducted on melanoma cell lines, it has been demonstrated that maintaining the M2 profile of TAMs depends on a high glycolytic flux [80].

PIM (Proviral Integration site for Moloney murine leukemia virus) proteins are members of a family of serine/threonine kinases that act downstream of cytokine receptors and are crucial for various aspects of cellular processes, including signal transduction, cell cycle progression, apoptosis, and cellular metabolism. Studies in animal models show that PIM blockade improves the function of T lymphocytes by inhibiting their switch to glycolytic metabolism and preventing terminal differentiation, thus favoring the central memory phenotype [81]. Additionally, studies conducted in a group of patients diagnosed with cHL suggest that lymphocytes remaining at earlier stages of development, without differentiating into more terminal forms, are associated with a favorable response to PD-1 blockade [82]. This combination approach represents a promising strategy for simultaneously targeting cancer cell metabolism and enhancing immune function.

### 4.2. Targeting Glucose Transporters

Another approach to disrupt glucose metabolism in cHL is by targeting glucose transporters, particularly GLUT1, which is often overexpressed in HRS cells. Small-molecule inhibitors of GLUT1, such as WZB117 and BAY-876, have shown promise in preclinical models of various cancers, including lymphomas [83,84]. While specific data on their efficacy in cHL is limited, these compounds represent a potential avenue for future research.

### 4.3. Lactate Dehydrogenase Inhibition

Lactate dehydrogenase A (LDHA), a key enzyme in the final step of aerobic glycolysis, has emerged as another potential target in HL. LDHA inhibitors, such as FX11 and Galloflavin, have demonstrated anti-tumor effects in preclinical models of various cancers [85]. Although their specific efficacy in cHL remains to be fully elucidated, these compounds could potentially disrupt the glycolytic phenotype of HRS cells and reduce lactate production in the TME, which has an immunosuppressive effect on TME cells [86].

### 4.4. Altering Mitochondrial Metabolism

Mitochondrial metabolism is another area of interest for therapeutic targeting in cHL. Inhibitors of oxidative phosphorylation, such as IACS-010759, which targets complex I of the electron transport chain, have shown efficacy in preclinical models of hematologic malignancies [87]. These agents may be particularly effective against cHL cells that have developed resistance to glycolysis inhibitors through metabolic plasticity. Moreover, inhibitors of mitochondrial metabolism, such as metformin or novel agents targeting the electron transport chain, have shown promise in preclinical studies and may synergize with glycolysis [88].

## 5. Exploiting Fatty Acid and Amino Acid Metabolism in cHL

Metabolic reprogramming, particularly involving the metabolism of fatty acids and amino acids, has emerged as a critical hallmark of cancer cell adaptation and survival. In cHL, increasing attention has been directed towards lipid and amino acid metabolic pathways, not only for their role in sustaining tumor proliferation but also in modulating the TME and contributing to therapy resistance [47,48].

### 5.1. Disrupting Lipid-Fueled Tumor Growth

Although direct evidence for targeting fatty acid metabolism in cHL remains limited, insights from other malignancies suggest promising therapeutic potential. Inhibition of fatty acid β-oxidation (FAO), particularly through the pharmacological blockade of CPT1, can reduce the energy supply, induce metabolic stress, and modulate immune responses within the TME [13,89].

Elevated plasma levels of elaidic acid in cHL patients—possibly influenced by dietary or systemic metabolic alterations—suggest a reprogramming of lipid metabolism that may represent a therapeutic vulnerability [50]. One key metabolic adaptation is increased de novo lipogenesis (DNL), often driven by fatty acid synthase (FASN). Overexpression of FASN supports membrane biosynthesis, energy storage, and oncogenic signaling. While direct data in cHL are limited, evidence from NHL indicates that FASN facilitates malignant proliferation through enhanced nucleotide and protein synthesis. FASN inhibitors, such as C75, TVB-2640, and orlistat, have demonstrated antiproliferative and pro-apoptotic effects in preclinical models [90].

In addition to lipid synthesis, cHL cells may utilize FAO as an alternative energy source. Inhibiting FAO with agents like etomoxir (targeting CPT1) may disrupt energy metabolism and induce tumor cell death. Moreover, blocking fatty acid uptake through transporters such as CD36 could deprive tumor cells of essential substrates [13].

Collectively, targeting fatty acid metabolism—via inhibition of FASN, FAO, or lipid transport—offers a compelling therapeutic strategy in cHL and warrants further investigation in both preclinical and clinical settings.

### 5.2. Starving Tumors of Critical Amino Acids

Metabolomic profiling in cHL has revealed significant alterations in purine and amino acid metabolism, reflecting the heightened proliferative and biosynthetic demands of HRS cells. Elevated plasma levels of hypoxanthine—a purine degradation product—likely indicate accelerated nucleotide turnover. While not specific to cHL, these findings support the rationale for targeting purine biosynthesis, such as with inosine monophosphate dehydrogenase (IMPDH) inhibitors, particularly in metabolically active or treatment-resistant cases [50,91].

HRS cells rely on increased amino acid uptake to sustain rapid growth and adaptation within the TME. The overexpression of amino acid transporters—including ASCT2 (SLC1A5) and LAT1 (SLC7A5)—facilitates glutamine and essential amino acid influx, fueling biosynthesis and mTORC1 signaling [13]. The pharmacological blockade of these transporters (e.g., GPNA, V-9302) or inhibition of glutaminase (GLS), a key enzyme in glutamine catabolism (e.g., CB-839), has demonstrated anti-tumor efficacy in lymphoma models [47,91].

Furthermore, the hexosamine biosynthetic pathway (HBP), which integrates glucose and glutamine metabolism, has emerged as a potential target for intervention. HBP inhibition may disrupt NF-κB signaling, a critical survival pathway in cHL cells [13].

Arginine metabolism also represents a promising target. Arginine-depleting therapies, such as pegylated arginine deiminase (PEG-ADI), may be effective in lymphomas with low argininosuccinate synthetase (ASS1) expression [47]. Similarly, the tryptophan catabolic enzyme indoleamine 2,3-dioxygenase (IDO) contributes to immune suppression within the cHL TME. IDO inhibitors, including epacadostat and indoximod, are currently under clinical investigation in various cancers, including lymphomas, to restore anti-tumor immunity [47].

Combination strategies targeting multiple metabolic pathways are also under exploration. For instance, co-inhibition of glycolysis and glutaminolysis has shown synergistic effects in preclinical studies, offering a rationale for dual metabolic targeting in cHL [92].

## 6. Immunometabolism-Based Therapies

The field of immunometabolism, which explores the interplay between cellular metabolism and immune function, has opened new avenues for cancer therapy. In the context of cHL, several immunometabolism-based approaches are being investigated to enhance anti-tumor immunity and improve treatment outcomes. A summary of selected therapeutic agents and their mechanisms of action is presented in Table 1, which categorizes immunometabolism-based approaches into preclinical studies (Table 1A) and clinical trials (Table 1B). This table highlights a broad range of strategies, from metabolic modulators like metformin and rapamycin to CPIs and cellular therapies, providing a comprehensive overview of the current therapeutic landscape.

### 6.1. Immunometabolic Modulators

Experimental data indicate that targeting key metabolic regulators, such as AMP-activated protein kinase (AMPK) and the mechanistic target of rapamycin (mTOR), may profoundly influence T-cell fate decisions. A study by Ara et al. demonstrated that the activation of AMPKα1 is essential for the rapamycin-mediated inhibition of the mTORC1–S6K signaling pathway, which is critical for enhancing memory T-cell generation. Specifically, AMPK activation promotes mitochondrial biogenesis and oxidative phosphorylation, favoring the development of CD8**^+^** memory T cells over terminally differentiated effector T cells. Conversely, hyperactivation of mTORC1–S6K signaling skews T-cell differentiation toward a glycolytic, short-lived effector phenotype, thereby limiting their persistence and anti-tumor capacity.

These findings provide a strong rationale for therapeutic strategies aimed at modulating T-cell metabolism—either through direct pharmacologic activation of AMPK or inhibition of mTORC1—to enhance the metabolic fitness and memory potential of tumor-reactive T cells. Such approaches could synergize with immune checkpoint blockade or adoptive T-cell therapies in the treatment of cHL and other malignancies, particularly in the context of an immunosuppressive or metabolically hostile TME [93].

Small-molecule activators of AMPK, such as metformin, have gained attention for their ability to improve anti-tumor immunity by modulating T-cell exhaustion mechanisms. Zhang et al. demonstrated that metformin enhances the cytotoxic activity of CD8**^+^** T cells through the AMPK–miR-107–Eomes–PD-1 axis, leading to a reduced expression of the inhibitory receptor PD-1 and improved effector function. Additionally, metformin-treated T cells exhibited superior mitochondrial fitness and cytokine production, further supporting the concept of metabolic reprogramming as a complementary approach to immunotherapy in cancer, including lymphomas [94].

Another promising strategy in cancer immunotherapy focuses on targeting metabolic checkpoints that regulate the immunosuppressive microenvironment. Among them, the ectonucleotidases CD39 and CD73 play a crucial role by catalyzing the conversion of extracellular ATP to adenosine, a potent immunosuppressive metabolite within the TME. The accumulation of adenosine contributes to T-cell dysfunction, promotes regulatory T-cell (Treg) activity, and facilitates immune escape mechanisms.

Recent preclinical data by Kolbe et al. demonstrated that pharmacological inhibition of the CD39/CD73 axis effectively restores anti-tumor immunity in nodal B-cell lymphomas. Importantly, the blockade of these enzymes synergized with bispecific anti-CD20 antibodies, enhancing the cytotoxicity of both T cells and natural killer (NK) cells [95]. Although these findings derive from NHL models, the adenosine pathway is also biologically relevant in cHL, where the TME is enriched with immunoregulatory molecules. Notably, a retrospective study by Grund et al. showed that CD73-low expression in cHL correlated with a significantly higher rate of relapsed/refractory (R/R) disease and shorter progression-free survival, suggesting CD73 as an independent negative prognostic marker. However, clinical trials evaluating CD39/CD73 inhibitors in cHL are currently lacking, highlighting the need for translational studies to explore this axis as a potential therapeutic target [95,96].

**Figure 2:**
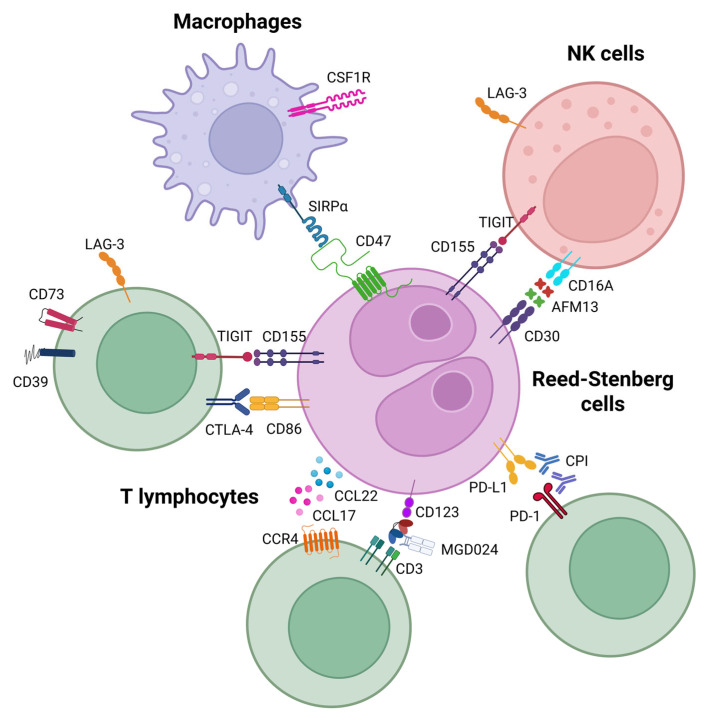
Selected immunotherapeutic targets and cellular interactions in cHL. LAG-3—Lymphocyte activation gene-3; CD73—ecto-5′-nucleotidase; CD39—ectonucleoside triphosphate diphosphohydrolase-1; TIGIT—T-cell immunoreceptor with Ig and ITIM domains; CD155—poliovirus receptor (PVR), a ligand for TIGIT; CTLA-4—cytotoxic T-lymphocyte antigen-4; CCL22—chemokine (C-C motif) ligand 22; CCL17—chemokine (C-C motif) ligand 17; CCR4 C—C chemokine receptor type 4; MGD024—a bispecific antibody targeting CD123 and CD3; CPI—checkpoint inhibitor; PD-1—programmed cell death protein 1; PD-L1—programmed death-ligand 1, a ligand for PD-1; CSF1R—colony-stimulating factor 1 receptor; SIRPα—signal regulatory protein alpha; AFM13—bispecific antibody construct targeting CD30 and CD16A; and CD47—a transmembrane protein that interacts with SIRPα.

Targeting the metabolic plasticity of myeloid cells in the TME represents a novel therapeutic concept in cHL. The inhibition of the colony-stimulating factor 1 receptor (CSF1R), a key regulator of macrophage differentiation, has been shown in preclinical studies to modulate the immunosuppressive landscape of various tumors. As reported by Cannarile et al., the pharmacological blockade of CSF1R can remodel TAMs, promoting their pro-inflammatory reprogramming and enhancing anti-tumor immune responses. These findings support the rationale for combining CSF1R inhibitors with other immunotherapies in cHL to overcome resistance driven by myeloid cells within the TME [97]. Figure 2 provides a comprehensive visual overview of these immunoregulatory pathways and cellular interactions within the cHL TME. The figure illustrates the complex network of checkpoint molecules (PD-1/PD-L1, CTLA-4, LAG-3, TIGIT), chemokine signaling (CCL17/CCL22-CCR4), and therapeutic targets including bispecific antibodies (AFM13, MGD024) and the CD47-SIRPα axis, highlighting the multiple intervention points available for immunometabolic targeting.

### 6.2. Epigenetic Modulators

Another emerging therapeutic strategy in cHL involves the use of epigenetic modulators, particularly inhibitors of enhancer of zeste homolog 2 (EZH2), which play a key role in regulating gene expression through chromatin remodeling. EZH2 is the catalytic subunit of the polycomb repressive complex 2 (PRC2), responsible for the trimethylation of histone H3 at lysine 27 (H3K27me3), leading to the transcriptional silencing of tumor suppressor genes. Aberrant EZH2 activity has been implicated in lymphomagenesis and resistance mechanisms in several lymphoid malignancies, including cHL.

SHR2554 is a selective EZH2 inhibitor evaluated in a phase 1, first-in-human, dose-escalation, dose-expansion, and clinical expansion trial in patients with R/R mature lymphoid neoplasms, including cHL [98]. The study enrolled patients across multiple dose levels to determine the maximum tolerated dose and recommended phase 2 dose. The inhibition of EZH2 activity by SHR2554 resulted in decreased levels of H3K27me3, reactivation of silenced genes, and inhibition of tumor cell proliferation. Clinical responses were observed irrespective of the presence of EZH2 mutations, with manageable safety profile characterized by primarily grade 1–2 adverse events. This supports the potential role of epigenetic modulation beyond genetically defined subsets [98].

Moreover, combining epigenetic modulators with CPIs has emerged as a promising approach to overcome resistance to PD-1 blockade in cHL. In a phase 1 study, the combination of nivolumab with CC-486 (oral azacitidine, a DNA methyltransferase inhibitor) demonstrated clinical activity in patients with PD-1-refractory cHL, with encouraging response rates and acceptable toxicity profile, suggesting that epigenetic therapy may restore sensitivity to immunotherapy by modifying the TME and enhancing antigen presentation [99].

Collectively, these findings highlight the therapeutic potential of epigenetic modulators, both as monotherapy and in combination with CPIs, particularly in the treatment of R/R cHL.

### 6.3. Checkpoint Inhibitors

Recent advances in molecular profiling and TME characterization have led to the proposal of a novel biologically driven genomic classification of cHL, distinguishing three distinct subtypes: inflammatory immune escape cHL, virally driven cHL, and oncogene-driven cHL This classification integrates genomic alterations, immune landscape, and clinical presentation, offering a new framework for the biological stratification of cHL. Importantly, it provides a rationale for therapeutic personalization based on tumor biology and microenvironmental features. The inflammatory immune escape cHL subtype is characterized by frequent copy number alterations, including amplification of PD-L1 locus at 9p24.1, and an immunosuppressive TME enriched in Tregs and follicular helper T cells (TFH), making it a particularly attractive target for CPIs. In this context, the PD-1/PD-L1 axis plays a key role in immune evasion in cHL. Monoclonal antibodies, such as nivolumab and pembrolizumab, block this interaction, thereby restoring T-cell function and enhancing anti-tumor immunity. In contrast, oncogene-driven cHL demonstrates a high tumor mutational burden, recurrent driver mutations (e.g., TNFAIP3, SOCS1), and a relatively immune-cold TME, suggesting the potential for treatment de-escalation in selected patients without compromising efficacy. Finally, virally driven cHL, strongly associated with Epstein–Barr virus (EBV) or human herpesvirus 6 (HHV6) infection, exhibits an inflammatory TME dominated by myeloid and cytotoxic cells, highlighting possible opportunities for immunomodulatory strategies. This biologic classification, therefore, represents a promising tool for individualized risk stratification and may inform future clinical trials exploring tailored treatment approaches in cHL [100].

The role of CPIs like nivolumab or pembrolizumab in the treatment of cHL is already well established. However, numerous ongoing clinical trials aim to improve treatment outcomes further and advance therapy personalization. These efforts extend beyond patients with R/R disease, increasingly focusing on the frontline setting, intending to minimize the use of radiotherapy and conventional chemotherapy by integrating novel, targeted therapeutic approaches [101].

Interestingly, the study by Rossi et al. suggests that prior exposure to PD-1 inhibitors may induce a resensitization effect, potentially restoring chemosensitivity in patients with R/R cHL who had previously failed conventional therapies. The analysis of patients treated across LYSA centers demonstrated that subsequent chemotherapy, administered after progression on anti-PD-1 therapy, achieved unexpectedly high response rates, including complete remissions, despite prior chemoresistance. These findings support the hypothesis that PD-1 blockade may modulate the TME or alter immune–tumor interactions in a manner that enhances the efficacy of subsequent cytotoxic treatment. This observation provides a strong rationale for further exploration of sequential or combination strategies integrating immunotherapy and chemotherapy in the management of R/R cHL [102].

Combining metabolic modulators with CPIs is an emerging strategy to overcome resistance mechanisms in cHL. The inhibition of glycolysis using 2-DG has been shown to enhance the efficacy of PD-1 blockade by reducing lactate-mediated immunosuppression and downregulating PD-L1 expression via inhibition of the pyruvate dehydrogenase kinase (PDK) pathway [103]. Additionally, recent clinical data suggest that inhibiting the JAK-STAT signaling pathway may further enhance responses to checkpoint blockade in cHL, likely through modulation of PD-L1 expression and the inflammatory microenvironment within the TME. These findings support the rationale for combining metabolic interventions or JAK inhibitors with immunotherapy in future therapeutic strategies for cHL [103,104].

In addition to PD-1/PD-L1 blockade, increasing attention in cHL research has focused on novel CPIs targeting alternative pathways of tumor immune escape. Among these, CTLA-4 blockade has emerged as a promising therapeutic strategy in cHL. Unlike PD-1, which predominantly marks exhausted T cells, CTLA-4 expression in cHL identifies a distinct population of non-regulatory (non-Treg) T cells within the TME. Patel et al. demonstrated that CTLA-4-positive, PD-1-negative T cells are highly enriched in cHL tissues, often outnumbering PD-1-expressing cells and preferentially localizing near HRS cells, which express CD86, the ligand for CTLA-4 [8]. This CTLA-4:CD86 interaction forms a direct immunosuppressive axis that contributes to immune evasion, which appears particularly prominent in R/R cHL, including tumors previously exposed to PD-1 inhibitors. These findings suggest that CTLA-4-mediated suppression may represent an adaptive resistance mechanism. Preclinical data indicate that targeting CTLA-4 can disrupt this interaction, restore T-cell activation, and enhance anti-tumor immunity, supporting the exploration of CTLA-4 inhibitors as a potential therapeutic option, particularly in PD-1-refractory cHL [8]. Early clinical data are emerging; in the CheckMate 039 study, the combination of nivolumab and ipilimumab achieved an overall response rate (ORR) of approximately 74% in R/R cHL, although with increased grade 3–4 toxicity [105]. Furthermore, ipilimumab monotherapy has demonstrated activity in patients with cHL relapsing after allogeneic stem cell transplantation [106] and ongoing clinical trials are investigating the role of ipilimumab ± nivolumab in heavily pretreated cHL (NCT04938232) [106,107]. These findings underscore the potential of CTLA-4 blockade as part of future therapeutic strategies in cHL.

Beyond CTLA-4, other emerging immune checkpoints under investigation in cHL include TIGIT (T-cell immunoreceptor with Ig and ITIM domains), LAG-3 (lymphocyte-activation gene 3), and CD47. Early-phase clinical trials have demonstrated encouraging activity of dual checkpoint blockade strategies, particularly in patients with R/R disease resistant to prior PD-1 blockade.

TIGIT is an inhibitory receptor expressed on T cells and NK cells. Its interaction with its ligand, CD155, present on tumor cells and antigen-presenting cells, leads to the suppression of T-cell activation and NK cell-mediated cytotoxicity. The blockade of TIGIT aims to restore anti-tumor immunity by enhancing both T-cell and NK-cell responses. In a phase 2 study, the combination of vibostolimab (anti-TIGIT antibody) with pembrolizumab showed preliminary clinical efficacy with an overall response rate and manageable safety profile in patients with R/R hematologic malignancies, including cHL. However, specific response rates and detailed safety data for the cHL cohort require further analysis [108].

Similarly, LAG-3 is an immune checkpoint receptor expressed on activated T cells, Tregs, and NK cells. LAG-3 binding to its ligands, including MHC class II molecules, negatively regulates T-cell proliferation and cytokine production. Favezelimab, an anti-LAG-3 antibody, in combination with pembrolizumab, demonstrated promising activity in patients with heavily pretreated, anti-PD-1-refractory classical cHL in early-phase clinical studies. The combination showed encouraging response rates in this heavily pretreated population, though detailed efficacy and safety data are still being evaluated [109].

Another potential therapeutic strategy in cHL involves targeting the CCL17/CCL22-CCR4 axis within the TME. HRS cells secrete the chemokines CCL17 (also known as thymus and activation-regulated chemokine, TARC) and CCL22, which recruit CCR4-expressing Th2 cells and Tregs, promoting an immunosuppressive milieu that facilitates tumor survival. This process is reinforced by the IL-4-mediated activation of the JAK/STAT6 pathway, which further drives CCL17 expression and Treg accumulation. Therapeutic approaches aimed at disrupting this immunoregulatory axis—such as the monoclonal anti-CCR4 antibody mogamulizumab and small-molecule CCR4 antagonists including FLX475 and CCR4-351—have shown promise in restoring anti-tumor immunity. These agents are being investigated for their potential to remodel the TME and enhance the effectiveness of standard chemotherapy and immune checkpoint blockade [110].

Particular attention has also been paid to the CD47-SIRPα axis, which regulates innate immune evasion. CD47, overexpressed on tumor cells, interacts with SIRPα on macrophages, delivering a “do not eat me” signal that inhibits phagocytosis. The blockade of CD47 disrupts this interaction, promoting macrophage-mediated clearance of tumor cells [111]. Recent clinical evidence supports the potential of this approach in cHL. In a phase II trial, magrolimab (anti-CD47 antibody) combined with pembrolizumab demonstrated an overall response rate (ORR) of 75% (three complete and three partial responses among eight patients), with a manageable safety profile—mostly low-grade (G1–2) anemia, hepatotoxicity, and lymphopenia [112]. This study, registered as NCT04788043, is ongoing to further evaluate efficacy and safety [113]. Collectively, these findings highlight the therapeutic promise of targeting the CD47-SIRPα axis, particularly when combined with PD-1 blockade, to enhance anti-tumor immunity and overcome resistance mechanisms in cHL [111,112,113].

### 6.4. Bispecific Antibodies and Bifunctional Fusion Proteins

Another innovative therapeutic approach in cHL involves the use of bispecific antibodies and bifunctional fusion proteins, which represent a promising class of immunotherapeutic agents currently under investigation. These molecules are designed to enhance anti-tumor immunity by simultaneously targeting tumor-associated antigens or by combining immune checkpoint blockade with modulation of the TME, aiming to overcome resistance mechanisms and improve treatment outcomes, particularly in R/R disease.

One of the most extensively studied bispecific antibodies in cHL is AFM13, which targets CD30, highly expressed on HRS cells, and CD16A on natural killer (NK) cells, facilitating antibody-dependent cellular cytotoxicity (ADCC). In a phase 1b clinical trial, patients with R/R cHL who received the combination of AFM13 with pembrolizumab demonstrated a high overall response rate (ORR) of 88% and a complete response (CR) rate of 46% in patients with R/R cHL, including those previously exposed to PD-1 inhibitors. The safety profile was manageable with treatment-related adverse events primarily consisting of infusion-related reactions and cytokine release syndrome, which were generally grade 1–2 [114].

Another promising approach involves bispecific antibodies targeting immune escape pathways. IBI322, a CD47/PD-L1 bispecific antibody, has been designed to simultaneously block innate and adaptive immune resistance mechanisms by inhibiting CD47-mediated phagocytosis resistance and PD-L1-driven T-cell suppression. Preliminary results from a phase 1 trial in patients with PD-1 or PD-L1 refractory cHL demonstrated favorable safety profile and encouraging clinical activity, though specific response rates and detailed safety data are pending full publication [115].

Additionally, bispecific antibodies targeting CD123, a marker expressed on HRS cells and components of the cHL TME, are under investigation. MGD024, a next-generation CD123 × CD3 molecule, is engineered to redirect cytotoxic T cells toward CD123-positive tumor cells while minimizing the risk of cytokine release syndrome (CRS) through optimized binding affinity and controlled T-cell activation [116]. In the ongoing first-in-human phase I clinical trial (NCT05362773), which enrolls patients with R/R hematologic malignancies including cHL, MGD024 has demonstrated an acceptable safety profile and early signs of efficacy across disease cohorts. However, detailed response rates and safety data specific to the cHL cohort have not yet been reported, and its clinical utility in cHL remains to be established [116,117].

Moreover, bifunctional fusion proteins such as SHR-1701, which combine PD-L1 blockade with TGF-β neutralization, represent an innovative strategy aimed at modulating both immune checkpoints and the immunosuppressive TME. While early clinical data initially focused on solid tumors [118], recent phase I results indicate promising activity in pretreated cHL. In a dose-escalation/expansion study combining SHR-1701 with the EZH2 inhibitor SHR2554 (NCT04407741), 16 heavily pretreated cHL patients were evaluable: an objective response rate of 100% and complete remission in 7% were observed, with an acceptable safety profile [119,120]. These encouraging findings support the further exploration of SHR-1701-based regimens in R/R cHL [118].

### 6.5. Antibody–Drug Conjugates (ADCs)

Brentuximab vedotin (BV) is an antibody–drug conjugate consisting of an anti-CD30 monoclonal antibody linked to the cytotoxic agent monomethyl auristatin E (MMAE), which disrupts microtubule function and induces apoptosis in CD30-expressing cells. CD30 is highly expressed on HRS cells, making BV a key therapeutic option in cHL [121]. The pivotal phase 3 ECHELON-1 trial (*n* = 1334 patients) demonstrated that the combination of BV with AVD significantly improved 4-year progression-free survival (94.3% vs. 90.9%, HR 0.68, *p* = 0.035) compared to standard ABVD in patients with advanced-stage cHL. The safety profile showed increased rates of peripheral neuropathy (67% vs. 43%) but reduced pulmonary toxicity (2% vs. 7%) compared to ABVD. Further validating BV’s role in frontline therapy, the HD21 trial (*n* = 1482) showed PET-guided BrECADD outperformed eBEACOPP, achieving 94.3% 4-year progression-free survival with significantly lower treatment-related morbidity (42% vs. 59%, *p* < 0.0001) [121]. Subsequent studies confirmed the efficacy of BV in both the frontline setting and in R/R disease, including after autologous stem cell transplantation. Moreover, BV-based regimens are increasingly being explored in pediatric and adolescent cHL populations. Despite its clinical benefits, peripheral neuropathy remains a common adverse event associated with BV, often requiring dose modifications or treatment discontinuation [122,123,124,125].

### 6.6. CAR-T and CAR-NK Approaches

Finally, one of the most groundbreaking advances in the treatment of R/R cHL is the development of cellular immunotherapies based on CAR-T and CAR-NK. These approaches offer highly personalized strategies that bypass conventional mechanisms of immune escape [126].

CAR-T cells are genetically modified T lymphocytes designed to recognize tumor-specific antigens independently of HLA presentation. In cHL, CD30—a molecule uniformly expressed on HRS cells—has emerged as the most extensively studied target. Clinical trials evaluating anti-CD30 CAR-T cells have demonstrated ORR ranging from 43% to 72% in heavily pretreated R/R cHL patients across multiple studies. However, the median duration of response was limited (range of 2–9 months), and treatment-associated toxicities included CRS in 15–30% of patients, with most cases being grade 1–2 [126,127]. Recently, single-cell transcriptomic studies have identified CD86 as an additional promising CAR-T target due to its strong and selective expression on HRS cells and TAMs, providing a novel opportunity to counteract CTLA-4:CD86-driven immune suppression and enhance CAR-T efficacy [128].

More recently, CAR-NK cells have received attention as a promising alternative to CAR-T therapy, offering potential advantages, including improved safety profiles (reduced risk of CRS) and the possibility of off-the-shelf application. In preclinical studies, CAR-NK cells targeting CD30 or CD70 demonstrated significant anti-lymphoma activity. Notably, CD70-specific CAR-NK cells engineered to express IL-15 showed enhanced proliferation, persistence, and cytotoxicity against lymphoma cells in vitro and in vivo models [129]. Nevertheless, limited trafficking of NK cells to lymphoid tissues remains a critical barrier to the effectiveness of CAR-NK therapies in lymphomas such as cHL. To overcome this challenge, Sanz-Ortega et al. demonstrated that engineering NK cells to express CCR7—a chemokine receptor involved in lymph node homing—significantly improved their migration to lymphoid compartments and enhanced anti-lymphoma activity [130]. Preliminary data from the ongoing phase 1/2 LuminICE-203 clinical trial (NCT05883449) combining allogeneic NK cells with AFM13 achieved an ORR of 86% and a CR rate of 55% in heavily pretreated patients with R/R cHL (*n* = cohort size pending full publication). The safety profile appeared favorable with reduced CRS risk compared to CAR-T approaches [131]. These findings highlight the potential of combining CAR-NK cell therapy with strategies aimed at improving tumor site localization to optimize therapeutic efficacy in cHL.

**Table 1:** Immunometabolism-based therapies.

(A) Preclinical Studies		
Therapeutic Modality	Mechanism of Action	References
AMPK activator—metformin	Activates AMPK pathway; reduces PD-1 expression; enhances effector function	[94]
mTOR inhibitor—rapamycin	Inhibits mTORC1–S6K pathway; promotes CD8^+^ memory T-cell differentiation	[93]
CD39/CD73 inhibitors	Inhibit adenosine production; restore T-cell and NK cell cytotoxicity	[95,96]
CSF1R inhibitors	Reprogram TAMs; enhance inflammatory and anti-tumor immune responses	[97]
Glycolysis inhibitor—2-deoxy-D-glucose (2-DG)	Reduces lactate-mediated immunosuppression; downregulates PD-L1 expression	[103]
Anti-CD70 CAR-NK cells with IL-15 expression	CD70-directed cytotoxicity enhanced by IL-15-mediated NK-cell proliferation and persistence	[129]
Anti-CD86 CAR-T-cell therapy	Targets CD86 on HRS cells and TAMs to overcome CTLA-4:CD86 immune suppression	[128]
**(** **B) Clinical Trials**		
**Therapeutic Modality**	**Mechanism of Action**	**References**
EZH2 inhibitor—SHR2554	Reduces H3K27me3 levels; reactivates silenced tumor suppressor genes; inhibits tumor proliferation	[98]
PD-1 inhibitors—nivolumab, pembrolizumab	Block PD-1 receptor on T cells; restore—cell activity and cytotoxicity by overcoming immune exhaustion in the TME	[100,101]
DNMT inhibitor—CC-486 + nivolumab	Reverses PD-1 resistance by modifying TME and enhancing antigen presentation	[99]
JAK-STAT pathway inhibitor	Modulates PD-L1 expression and inflammatory milieu in TME; enhances PD-1 blockade efficacy	[104]
CTLA-4 inhibitor	Disrupts CTLA-4:CD86 immunosuppressive axis; restores T-cell activation	[8,105,107]
TIGIT inhibitor—vibostolimab	Blocks TIGIT–CD155 interaction; enhances T- and NK-cell responses	[108]
LAG-3 inhibitor—favezelimab	Boosts T-cell proliferation and cytokine production	[109]
CCR4-targeting agents—mogamulizumab, FLX475, CCR4-351	Blocks Treg recruitment via CCL17/CCL22–CCR4 axis; restores anti-tumor immunity	[110]
CD47 inhibitor	Disrupts CD47-SIRPα interaction; promotes macrophage-mediated phagocytosis	[111,112,113]
Bispecific antibody—AFM13 (CD30/CD16A)	Mediates NK-cell ADCC against HRS cells	[114]
Bispecific antibody—IBI322 (CD47/PD-L1)	Blocks innate and adaptive immune evasion by inhibiting CD47 and PD-L1; promotes phagocytosis and T-cell activation	[115]
Bispecific antibody—MGD024 (CD123 x CD3)	Redirects T cells to CD123-expressing tumor cells	[116,117]
Bifunctional fusion protein—SHR-1701 (PD-L1 + TGF-β)	Simultaneous blockade of PD-L1 and TGF-β pathways; modulates immunosuppressive TME	[119,120]
Antibody–drug conjugate—brentuximab vedotin (BV)	Anti-CD30 antibody linked to MMAE; induces apoptosis in HRS cells	[122,123,124]
Anti-CD30 CAR-T-cell therapy	HLA-independent CD30 recognition and T cell-mediated cytotoxicity	[126,127]

## 7. Conclusions and Outlook

cHL exemplifies the complexity of tumor–host interactions, where a minority population of HRS cells orchestrates a metabolically active and immunosuppressive TME. Recent advances have highlighted the central role of tumor and immune cell metabolism in disease pathogenesis, progression, and treatment resistance. HRS cells exhibit significant metabolic reprogramming, including enhanced glycolysis, glutaminolysis, and lipid utilization, which not only fuels tumor growth but also modulates the immune landscape, contributing to immune evasion.

Therapeutic strategies that target metabolic vulnerabilities—such as glycolysis inhibitors, fatty acid metabolism blockers, and modulators of amino acid pathways—offer promising avenues for treatment, particularly when integrated with immune checkpoint blockade. Novel agents, such as epigenetic modulators, bispecific antibodies, and CAR-based cellular therapies, continue to expand the therapeutic armamentarium, particularly for R/R cHL.

Future research should prioritize several specific and targeted investigations to advance the field, as follows:

First, mechanistic studies are needed to elucidate the precise metabolic crosstalk between HRS cells and specific TME components. Priority areas include: (1) detailed characterization of the lactate shuttle between glycolytic stromal cells and oxidative HRS cells, including identification of specific monocarboxylate transporter expression patterns and their therapeutic targeting; (2) investigation of lipid metabolism reprogramming in cHL, particularly the role of fatty acid β-oxidation in HRS cell survival and its interaction with immune cell metabolism; and (3) comprehensive mapping of amino acid dependencies in HRS cells, with focus on glutamine addiction and arginine metabolism as therapeutic vulnerabilities.

Second, immune checkpoint research should expand beyond PD-1/PD-L1 to systematically evaluate emerging targets. Specific priorities include: (1) mechanistic studies of CTLA-4+ non-regulatory T cells in cHL and their interaction with CD86+ HRS cells; (2) investigation of LAG-3 and TIGIT co-expression patterns on tumor-infiltrating lymphocytes and their sequential or simultaneous targeting strategies; and (3) detailed characterization of the CD47-SIRPα axis in cHL macrophage biology and its modulation by metabolic interventions.

Third, translational research should focus on developing predictive biomarkers and patient stratification approaches: (1) validation of metabolic signatures (such as TMTV, lactate/pyruvate ratios, and amino acid profiles) as prognostic and predictive biomarkers; (2) development of functional metabolic assays to guide personalized combination therapy selection; and (3) investigation of epigenetic markers (particularly EZH2 activity and DNA methylation patterns) as determinants of immunotherapy response.

Fourth, combination therapy optimization requires systematic investigation of: (1) optimal sequencing and dosing of metabolic inhibitors with checkpoint blockade; (2) rational design of triple combinations targeting metabolism, immune checkpoints, and epigenetic regulation; and (3) development of biomarker-driven adaptive trial designs to personalize combination strategies.

Finally, pediatric and adolescent cHL research should specifically address: (1) age-specific metabolic differences in HRS cells and TME composition; (2) long-term effects of metabolic interventions on developing immune systems; and (3) optimization of CAR-T and CAR-NK approaches for younger patients with focus on minimizing late effects.

Ultimately, the integration of metabolic, immunologic, and epigenetic targeting represents a rational, multifaceted approach to improve clinical outcomes and reduce treatment-related toxicity. Future research should focus on developing precision medicine frameworks that incorporate metabolic profiling for patient stratification and therapy optimization. As our understanding of cHL metabolism and the TME deepens, these insights hold the potential to transform current treatment paradigms and achieve more durable and personalized therapeutic responses.

## Abbreviations

The following abbreviations are used in this manuscript:

2-DG	2-deoxy-D-glucose
AMPK	5′ adenosine monophosphate-activated protein kinase
BV	Brentuximab vedotin
CAR-NK	Chimeric antigen receptor-engineered natural killer cells
CAR-T	Chimeric antigen receptor-engineered T cells
cHL	Classical Hodgkin Lymphoma
CPIs	Immune checkpoint inhibitors
CPT-1	Carnitine palmitoyltransferase 1
CRS	Cytokine release syndrome
EBV	Epstein–Barr Virus
EZH2	Enhancer of zeste homolog 2
FAO	Fatty acid β-oxidation
FASN	Fatty acid synthase
HIF-1	Hypoxia-inducible factor 1
HRS	Reed–Sternberg cell
LDHA	Lactate dehydrogenase A
MCT	Monocarboxylate transporter
NHL	Non-Hodgkin lymphoma
NK	Natural Killer
ORR	Overall response rate
OXPHOS	Oxidative phosphorylation
PD-1	Programmed death receptor 1
PIM	Provirus Integration site for Moloney leukemia virus
TAMs	Tumor-associated macrophages
TIGIT	T-cell immunoreceptor with Ig and ITIM domains
TME	Tumor microenvironment
TOMM20	Translocase Of Outer Mitochondrial Membrane 20
Tregs	Regulatory T cells
